# Identifying Gastrointestinal Pathologies Using Point-of-Care Ultrasound

**DOI:** 10.3390/diagnostics16030418

**Published:** 2026-02-01

**Authors:** Rebecca G. Theophanous, Lior Abramson, Yuriy S. Bronshteyn

**Affiliations:** 1 Durham Veterans Affairs Healthcare System, Durham, NC 27710, USA; lior.abramson@duke.edu (L.A.); yuriy.bronshteyn@duke.edu (Y.S.B.); 2 Department of Emergency Medicine, Duke University School of Medicine, 2301 Erwin Rd, Durham, NC 27710, USA; 3 Department of Medicine, Duke University School of Medicine, Durham, NC 27710, USA; 4 Department of Anesthesiology, Duke University School of Medicine, Durham, NC 27710, USA

**Keywords:** bowel ultrasound, point-of-care ultrasound, appendicitis ultrasound, intussusception ultrasound, hernia ultrasound, abdominal mass ultrasound, diverticulitis ultrasound

## Abstract

Patients presenting with abdominal pain require expedited diagnosis and treatment. Computed tomography (CT) scans, which are frequently ordered in the inpatient and emergency departments, have high diagnostic sensitivity and specificity. However, CTs are costly, have radiation exposure, can create hospital workflow inefficiencies, and create a potential safety risk with patient transport. Point-of-care ultrasound (POCUS) use is growing as an efficient, safe, and bedside assessment tool for diagnosing and treating gastrointestinal (GI) pathologies. This manuscript synthesizes key sonographic findings and techniques for a series of important GI pathologies that physicians should recognize: diverticulitis, hernia, appendicitis, intussusception, and intra-abdominal mass.

**Figure 1:**
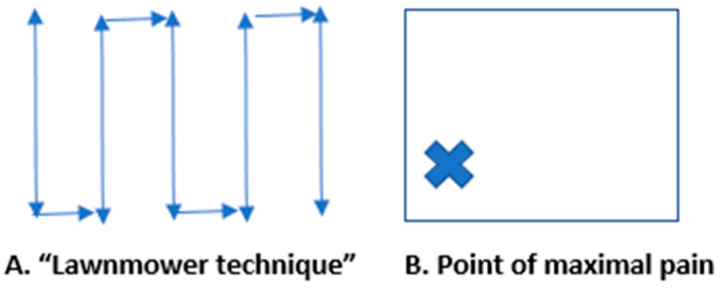
Abdominal pain is one of the most common presenting emergency department (ED) complaints [1,2,3,4]. It is also a frequent symptom that develops in hospitalized patients [4,5,6]. Computed tomography (CT) has traditionally been utilized for the diagnosis of gastrointestinal (GI) pathologies. However, CT has several limitations, including patient exposure to ionizing radiation, safety risks with transport of unstable patients, high costs, and delayed diagnostic times [2,3,4,7]. Consequently, recent guidelines from emergency medicine and critical care societies emphasize the role of POCUS as a first-line imaging tool in undifferentiated abdominal pain to guide management and reduce unnecessary CT scans [8,9,10]. Point-of-care ultrasound (POCUS) use is increasing for rapid bedside diagnosis of various pathologies, including GI disease [3,5,6,11,12]. Few studies in the literature have described the findings of gastrointestinal POCUS, which can improve patient outcomes through expedited inpatient and ED diagnosis and treatment [2,3,5,6]. This manuscript describes POCUS GI pathology findings with an illustrative series of ultrasound images for diverticulitis, hernia, appendicitis, intussusception, and intra-abdominal mass. We describe two techniques for performing a point-of-care abdominal ultrasound: (1) Place the curvilinear probe at the right upper quadrant in a transverse position and translate the probe inferiorly to the right lower quadrant, then slide 1–2 cm to the right and continue scanning the abdomen in vertical lines to visualize the entire abdomen and search for pathology. This is called the “lawnmower technique”. (2) Place the curvilinear ultrasound probe at the point of maximal pain and scan the area by (a) tilting the probe from superior to inferior while the probe is in a transverse position and then (b) tilting the probe from right to left with the probe in a longitudinal position. The user can slowly apply graduated pressure to displace bowel gas for improved viewing if needed. Normal small bowel (SB) measures < 3 cm in diameter, and large bowel (LB) measures < 6 cm. SB is defined by plicae circulares (Kerckring folds), which are bands of muscle along the SB wall, and LB is defined by haustra, which are wider outpouchings. Further, LB is located at the peripheral abdomen in the ascending, transverse, and descending positions, whereas SB is located centrally [2,3,5,6,7,11].

**Figure 2:**
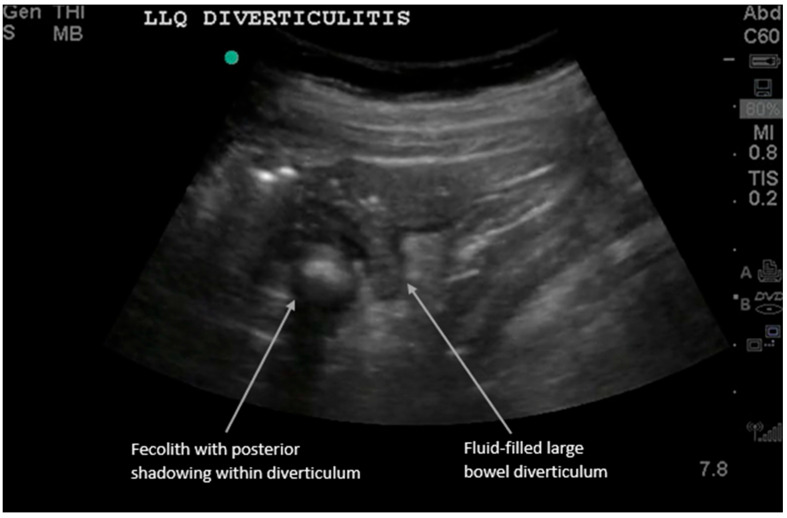
Acute diverticulitis results from inflammation of a colonic diverticulum and most commonly presents with left lower quadrant pain since it typically involves the descending or distal sigmoid colon. The sonographic diagnosis of diverticulitis, as detailed in recent comprehensive reviews, relies on visualizing diverticula as small outpouchings from the colon wall with posterior acoustic shadowing if gas or feces are present [2,5,13,14,15,16]. We present a long-axis view of large bowel diverticulitis, which has the following characteristics on ultrasound: (i) thickened large bowel wall > 5 mm, (ii) hyperemic bowel wall with color Doppler flow mode, (iii) fluid-filled contents in the diverticula, and (iv) non-compressible hyperechoic fat around the bowel. (See Supplementary Video S1 for examples of dynamic findings.) Sometimes the diverticulum can be visualized with a fecolith, and when this obstructs the diverticular neck, it can develop into diverticulitis. Most cases are uncomplicated (85%) and improve with nonoperative antibiotic treatment. However, complicated cases can show adjacent abscesses, fistulas, obstructions, or free air [6,11,12,13,14,15,16,17].

**Figure 3:**
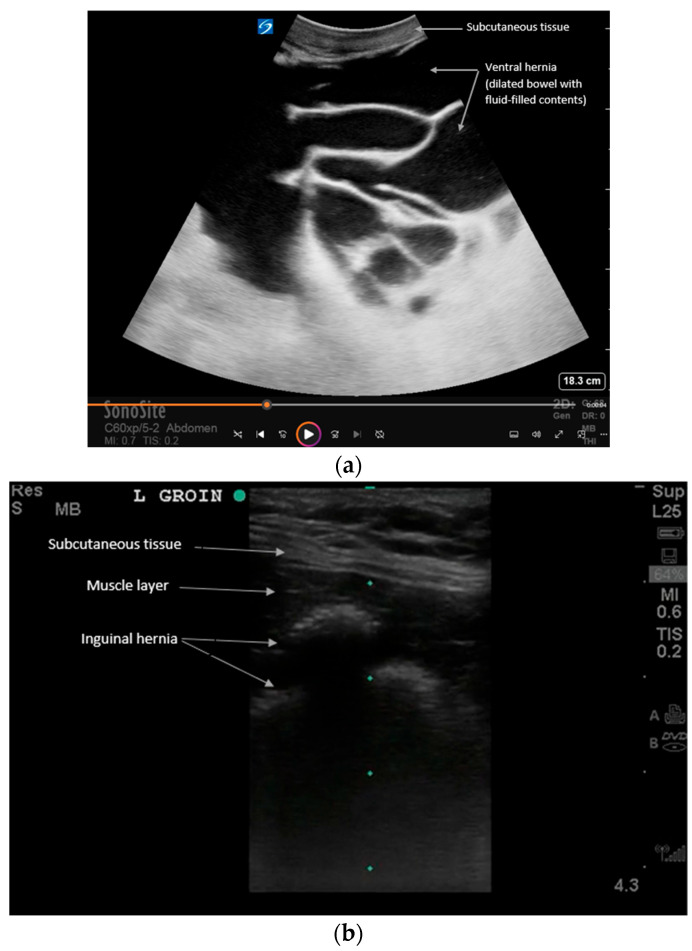
Examples of (**a**) ventral and (**b**) inguinal hernias that contain loops of bowel on POCUS. See Supplementary Videos S2 and S3 for examples of dynamic findings. Intestinal hernias can be evaluated with ultrasound at the bedside for faster recognition and manual reduction. Ultrasound reveals loops of bowel traversing the abdominal wall muscles. Dynamic assessment with Valsalva is recommended per expert consensus to evaluate reducibility and complications [11]. For example, the patient can be asked to perform a Valsalva maneuver to exaggerate the hernia during real-time ultrasound. Increased vascular flow, extra-luminal fluid, bowel dilation, or wall thickening can all suggest an increased risk for hernia incarceration or strangulation, which requires expedited treatment. Also, the lack of peristalsis within the bowel is a sign of complication [18].

**Figure 4:**
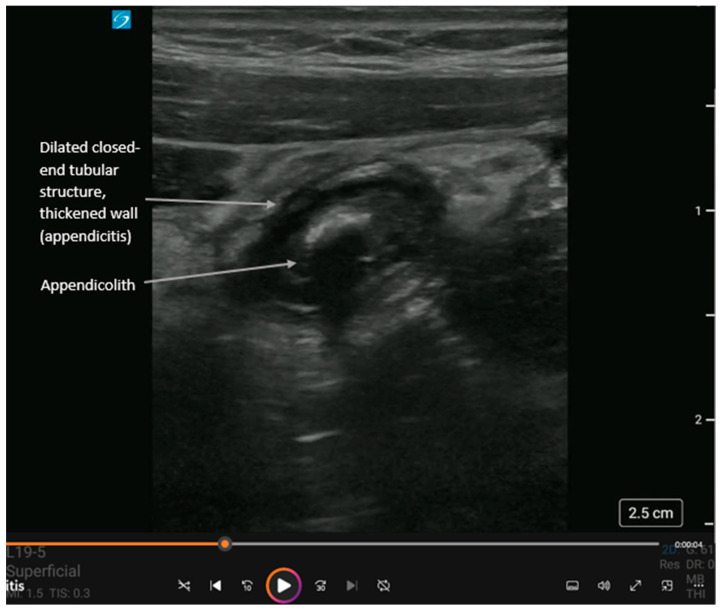
Short-axis view of acute appendicitis with an appendicolith and dynamic findings in Supplementary Video S4. The appendix is a closed-ended tubular structure usually found in the right lower quadrant of the abdomen. Landmarks for locating the appendix are the psoas muscle laterally and inferiorly, the external iliac vessels medially and inferiorly, and the internal oblique and transversus abdominis muscles anteriorly. In cases of acute appendicitis, the appendix diameter is dilated > 5 mm and non-compressible. Like with diverticulitis, ultrasound shows (i) thickened bowel wall > 3 mm, (ii) hyperemic bowel wall, and (iii) hyperechoic surrounding fat. An appendicolith (hyperechoic circular structure with posterior shadowing) or debris may be visualized within the bowel lumen. Also, complications can include extra-luminal free fluid (anechoic adjacent to the appendix), an abscess (heterogeneous collection adjacent to the appendix), or perforation (disruption of the appendiceal wall with either free fluid or abscess). While operator-dependent, recent meta-analyses report pooled sensitivity and specificity for POCUS diagnosis of appendicitis to be approximately 81% and 87%, respectively, making it a highly effective rule-in tool [19,20,21,22].

**Figure 5:**
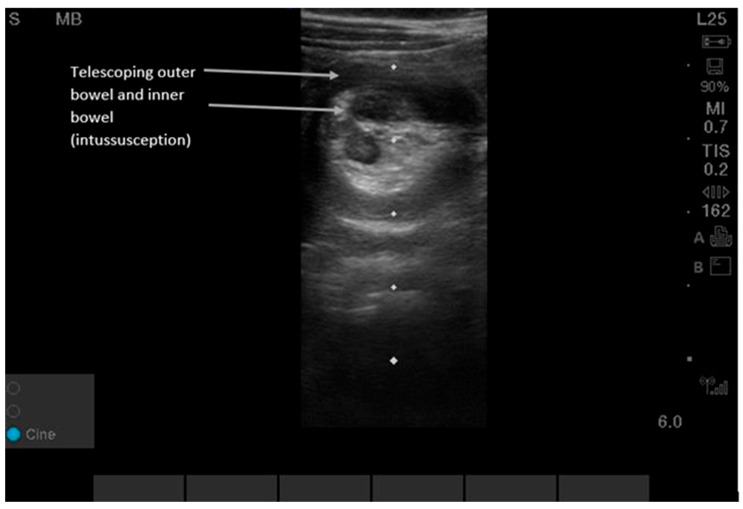
Intussusception can be detected by either the lawnmower or the point-of-maximal-pain technique. Users can begin in the right upper quadrant since this is the most common location (80% of intussusceptions are ileo-colonic) and then scan along the entire colon in the peripheral abdominal wall area using a “picture-frame” technique [23,24,25,26]. Supplementary Video S5 shows dynamic findings. POCUS shows a “target-shaped” or “donut-shaped” mass > 2 cm, with telescoping of one section of bowel into the adjacent bowel. Sensitivity and specificity for POCUS were 96.6% (95% CI, 82.2, 99.1) and 98% (95% CI 96.5, 99.9) in pediatric patients aged three months to six years old at 17 pediatric EDs in North and Central America, Europe, and Australia between 2018 and 2020 [23,24,25,26]. While the classic ‘target sign’ is consistent, intussusception in adults is frequently pathologic and requires identification of a potential lead-point mass, differing from the often idiopathic pediatric presentation [23,24,25,26].

**Figure 6:**
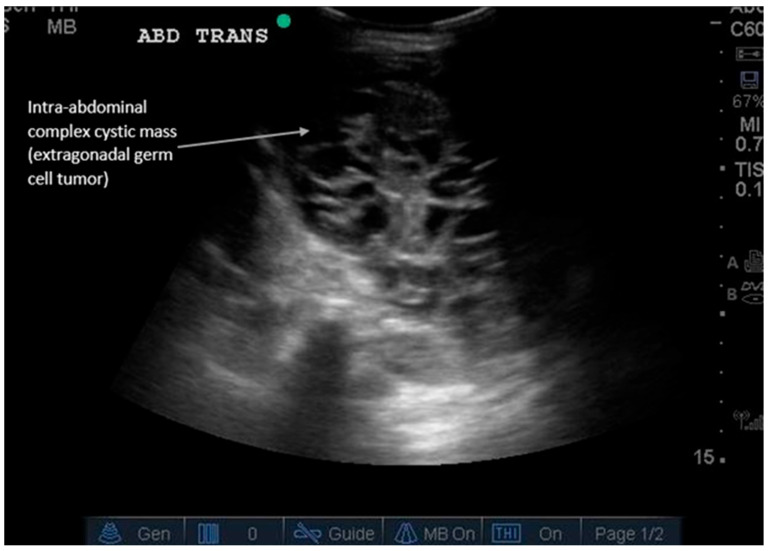
Incidental abdominal mass. Abdominal masses can present differently depending on their internal composition. Ultrasound is the first-line modality for palpable masses because it can be performed at the site of palpation, determine echogenicity, and guide biopsy for definitive diagnosis. Supplementary Video S6 shows dynamic findings of an extragonadal germ cell tumor presenting as a complex, cystic intra-abdominal mass in an infant. Masses can be solid (hyperechoic), cystic (anechoic), or fatty (e.g., lipomas); simple or complex (can have septations); or can contain calcifications (1–2 mm small hyperechoic structures with posterior shadowing). Adding color Doppler can evaluate vascular flow within the mass (positive flow is more suggestive of malignancy). A round vs. irregular shape also helps determine whether it is benign or malignant, respectively. Ultrasound is particularly useful in pediatric abdominal masses to avoid radiation from CT scans and allow expedited diagnosis. Types of pediatric malignant masses include Wilms’ tumor (typically intrarenal), hepatoblastoma (liver), neuroblastoma, non-Hodgkin’s lymphoma, germ cell tumor (ovarian or testicular), and others [27]. Subsequent imaging for detected masses includes Magnetic Resonance Imaging (MRI) for indeterminate fat-containing or solid masses and CT for fluid collections after trauma or surgery or for suspected metastases requiring restaging imaging [18,27]. Supplemental Table S1 summarizes the major diagnostic findings for each GI pathology described and the recommended locations for POCUS scanning. Supplemental Table S2 describes the sensitivity and specificity of ultrasound compared to computed tomography (CT) for various gastrointestinal pathologies [13,14,15,18,19,20,21,24,26,27,28]. Conclusions: In summary, POCUS is a validated, guideline-endorsed tool for the rapid initial assessment of common GI pathologies. Its integration into clinical pathways for abdominal pain can improve diagnostic efficiency, reduce radiation exposure, and expedite definitive care.

## Data Availability

The original contributions presented in this study are included in the article. Further inquiries can be directed to the corresponding author.

## Supplementary Materials

The following supporting information can be downloaded at https://www.mdpi.com/article/10.3390/diagnostics16030418/s1, Video S1: Acute diverticulitis with a fluid-filled diverticulum containing a fecolith with posterior shadowing. Video S2: Ventral hernia with dilated loops of bowel extending into the abdominal wall layers. Video S3: Inguinal hernia with bowel extending into the muscular abdominal wall layers. Video S4: Short-axis view of a dilated appendix containing an appendicolith with posterior shadowing. Video S5: Short-to-long-axis view of intussusception, with bowel telescoping into bowel appearing as a “donut-shaped” in short axis. Video S6: Complex cystic extragonadal germ cell tumor identified within the abdomen. Table S1: Major diagnostic findings for each GI pathology described and the recommended locations for POCUS scanning. Table S2: Sensitivity and specificity of ultrasound compared to computed tomography (CT) for various gastrointestinal pathologies.

## Abbreviations

The following abbreviations are used in this manuscript:

POCUS	Point-of-care ultrasound
GI	Gastrointestinal
ED	Emergency department
