# Supplementary material for: Identifying Gastrointestinal Pathologies Using Point-of-Care Ultrasound
Source: Diagnostics (Basel). 2026 Feb 1;16(3):418. doi: 10.3390/diagnostics16030418 (PMC12896783; doi:10.3390/diagnostics16030418)
Supplement: Supplementary file 1 [file diagnostics-16-00418-s001.zip › Table S1.pdf]

Supplemental Table S1. Major diagnostic findings for each GI pathology described and the recommended locations for POCUS scanning

Gastrointestinal pathology	Diagnostic findings on ultrasound	Anatomic region of interest	Most common location
Diverticulitis	Fluid-filled bowel small outpouchings, thickened wall, hyperemic wall, possible fecolith/adjacent fluid/abscess/perforation	Descending or distal sigmoid colon	Left lower quadrant
Hernia	Bowel extending into the abdominal wall, can be fluid-filled, hyperemic wall, with extra-luminal fluid, and lack of peristalsis	Ventral abdominal wall, inguinal canal, or scrotal sac	Abdominal wall, inguinal area (groin), or scrotum
Appendicitis	Dilated tubular closed-end structure >5mm, thickened wall, hyperemic wall, possible appendicolith/adjacent fluid/abscess/perforation	Adjacent to ileum	Right lower quadrant
Intussusception	"target-shaped" or "donut-shaped" bowel telescoping into bowel	Ileo-colic or colo-colic	Right upper quadrant
Abdominal mass	*Benign characteristics*: simple, anechoic, round or oval shape<br>*Malignant*: complex, hyperechoic, irregular borders, vascular flow, calcifications	Renal (Wilms), liver (hepatoblastoma), ovarian or testicular (germ cell tumor), various locations for other masses	Any location
