# Supplementary material for: Identifying Gastrointestinal Pathologies Using Point-of-Care Ultrasound
Source: Diagnostics (Basel). 2026 Feb 1;16(3):418. doi: 10.3390/diagnostics16030418 (PMC12896783; doi:10.3390/diagnostics16030418)
Supplement: Supplementary file 1 [file diagnostics-16-00418-s001.zip › Table S2.pdf]

Supplemental Table S2. Sensitivity and specificity of ultrasound compared to computed tomography (CT) for various gastrointestinal pathologies[13-15,18-21,24,26-28].

Gastrointestinal pathology	Ultrasound sensitivity	Ultrasound specificity	Contrast-enhanced CT sensitivity	Contrast-enhanced CT specificity
Diverticulitis	92-94% (95% CI: 0.92-0.95)	86-94% (95% CI: 0.72-0.94)	100%	100%
Hernia	98%	88%	70-85% with Valsalva, 54-80% without	92-98% with Valsalva, 25-65% without
Appendicitis	81-91% (95% CI: 0.78-0.96)	87-97% (95% CI: 0.85-0.99)	97% (95% CI: 0.95-0.98)	96% (95% CI: 0.94-0.97)
Intussusception	94-95% pediatric (95% CI: 0.90-0.97)	96-98% pediatric (95% CI: 0.96-0.99)	58-100% adults	57-71% adults
Abdominal mass	>95%	>95%	99%	97%
